# Pre-hospital rule-out of acute coronary syndrome by modified HEART score assessment including point-of-care troponin

**DOI:** 10.1007/s12471-022-01726-3

**Published:** 2022-10-19

**Authors:** C. Camaro, G. W. A. Aarts, N. van Royen, the ARTICA investigators

**Affiliations:** Department of Cardiology, Radboud University Medical Centre, Nijmegen, The Netherlands

Dear editor, with interest we read the results of the URGENT 1.5 trial in the article by Koper et al., recently published in the *Netherlands Heart Journal *[1]. The investigators retrospectively analysed 96 patients with chest pain, of whom 33 (34%) were diagnosed with acute coronary syndrome (ACS). The sensitivity and negative predictive value of the modified HEART score, which integrates fingerstick point-of-care (POC) troponin testing, were 97.0% and 97.6%, respectively. The authors point out that further prospective investigation of the applicability of the modified HEART score in a pre-hospital setting is needed.

We agree that further investigation of this topic should be encouraged. However, prospective studies on the applicability of the pre-hospital modified HEART score have already been performed [2, 3]. Although the POC troponin assay used in these studies was not a high-sensitivity assay nor a fingerstick, their results have shown that pre-hospital assessment of the modified HEART score with this assay is safe.

Moreover, the Dutch ARTICA trial (Acute Rule-Out of Non-ST-Segment Elevation Acute Coronary Syndrome in the (Pre)hospital Setting by HEART Score Assessment and a Single Point-of-Care Troponin; *ClinicalTrials.gov, NCT05466591*) addressed the above-mentioned issue [4]. In this investigator-initiated randomised controlled trial, we randomly allocated 866 patients with a modified HEAR score (i.e. HEART score without troponin component) ≤ 3 to: (a) standard emergency department admittance to rule out ACS or (b) POC troponin measurement and transfer of care to the general practitioner if troponin concentration was low. The primary outcome was healthcare costs at 30 days, and the secondary outcome consisted of major adverse cardiac events (all-cause mortality, ACS and unplanned revascularisation). Enrolment was completed on 4 May 2022, and the results were presented as a late-breaking clinical trial at the ESC Congress 2022 in Barcelona, Spain.

## References

[1] Koper LH, Frenk LDS, Meeder JG, van Osch FHM, Bruinen AL, Janssen MJW (2022). URGENT 1.5: diagnostic accuracy of the modified HEART score, with fingerstick point-of-care troponin testing, in ruling out acute coronary syndrome. Neth Heart J.

[2] Aarts GWA, van der Wulp K, Camaro C (2020). Pre-hospital point-of-care troponin measurement. Neth Heart J.

[3] Tolsma RT, Fokkert MJ, van Dongen DN (2022). Referral decisions based on a&nbsp;pre-hospital HEART score in suspected non-ST-elevation acute coronary syndrome: final results of the FamouS Triage study. Eur Heart J Acute Cardiovasc Care.

[4] Aarts GWA, Camaro C, van Geuns RJ (2020). Acute rule-out of non-ST-segment elevation acute coronary syndrome in the (pre)hospital setting by HEART score assessment and a&nbsp;single point-of-care troponin: rationale and design of the ARTICA randomised trial. Bmj Open.

